# Digital health interventions as a catalyst for change: A narrative review of the roles of digital health in enhancing well-being and reducing public health and social issues among sex workers

**DOI:** 10.1177/20552076251382056

**Published:** 2026-01-23

**Authors:** L. Winter Mokhwelepa, G. Olivia Sumbane

**Affiliations:** School of Medicine, Faculty of Health Sciences, 37714 University of Limpopo, Polokwane, South Africa

**Keywords:** Digital health, sex workers, well-being, public health, interventions

## Abstract

**Background:**

Digital health interventions (DHIs) are increasingly recognized for their potential to address public health and social issues. Among sex workers, these interventions have demonstrated promise in areas such as mental health support, harm reduction, and access to healthcare services. Importantly, the social and structural barriers faced by sex workers, including criminalization, stigma, and marginalization, are deeply intertwined with poor health outcomes, and DHIs may offer a means to tackle both simultaneously. However, existing literature often lacks a comprehensive understanding of their broader roles in enhancing overall well-being and addressing systemic challenges faced by this population, such as limited access to digital tools, inadequate integration of DHIs in sex work health services, and the absence of tailored digital strategies for marginalized groups. This review seeks to bridge this gap by examining the diverse applications and impacts of DHIs on the well-being of sex workers and their ability to mitigate public health and social issues.

**Objective:**

To review the roles of DHIs in enhancing well-being and reducing public health and social challenges among sex workers, while identifying key opportunities and barriers.

**Methods:**

A narrative review methodology was employed. Relevant peer-reviewed articles and gray literature published between 2010 and 2023 were identified through systematic searches of databases including PubMed, Scopus, Science Direct, CINAHL, Web of Science, and PsycINFO. The search used keywords such as “digital health,” “sex workers,” “public health,” “social well-being,” and “interventions.” Studies were included based on relevance, methodological rigor, and focus on sex workers. Inclusion criteria comprised studies that specifically addressed DHIs targeting sex workers, were published in English, and provided empirical findings or substantial discussions on well-being or public health outcomes. Exclusion criteria included articles that focused solely on general digital health tools without reference to sex workers, opinion pieces without empirical support, and studies outside the stated time frame. Data were charted and thematically analyzed to map trends, opportunities, and gaps.

**Results:**

Findings indicated that DHIs play a significant role in reducing systemic barriers that perpetuate poor health outcomes among sex workers. For example, mobile-based mental health platforms provided confidential support that helped overcome stigma and fear of discrimination in traditional healthcare settings. Digital harm reduction tools, such as online peer-education networks, were effective in reducing unsafe practices by addressing the marginalization that often prevents sex workers from accessing mainstream services. Furthermore, DHIs tailored to regions where sex work is criminalized enabled discreet access to sexual and reproductive health services, demonstrating how technology can mitigate the health risks tied to structural inequities. Nonetheless, gaps persist due to limited digital literacy, inadequate infrastructural support, and the absence of sex worker-centered policy frameworks.

**Conclusion:**

DHIs are a catalyst for change, addressing critical health and social disparities among sex workers. Recognizing the co-constitution of public health and social challenges enables a more integrated approach to intervention design and policy development. However, to maximize their impact, future efforts should focus on overcoming barriers, fostering inclusivity, and ensuring sustainability. Specific actions should include investing in digital infrastructure tailored to the needs of sex workers, co-developing interventions with community input, training healthcare providers on digital tools and sex worker-specific issues, and creating policies that protect digital privacy and reduce stigma. Policymakers and stakeholders must collaborate to integrate these interventions into broader public health frameworks.

## Introduction

Sex workers, a marginalized and often stigmatized group, face a range of health and social challenges that contribute to their vulnerability and disproportionately affect their well-being.^1–3^ These challenges are exacerbated by structural inequalities that uniquely affect sex workers more severely than other populations. For example, criminalization and fear of arrest often deter them from seeking timely medical care, unlike individuals in other professions who can access services without legal repercussions.^1^ Furthermore, these challenges include exposure to sexually transmitted infections (STIs), violence, mental health issues, substance abuse, and social exclusion.^1^ Moreover, many sex workers experience barriers to accessing healthcare services due to discrimination, stigmatization, and fear of legal repercussions.^1,3^ Policies and laws in some countries, particularly those that criminalize sex work, further exacerbate these barriers by creating an environment of fear and mistrust. Criminalization can discourage sex workers from seeking medical help, as doing so might expose them to arrest, police harassment, or forced disclosure of their occupation, leading to further punishment or abuse.^1^ In addition, intimate partner violence and image-based sexual abuse, both online and offline, pose significant threats to the health, safety, and mental well-being of sex workers, further compounding their marginalization.^1,2^ Consequently, there is a critical need to explore innovative strategies that can address these barriers and enhance the health and well-being of sex workers, particularly through the lens of digital health interventions (DHIs).

DHIs have emerged as a promising solution in addressing public health and social issues, offering a means of reaching sex workers more effectively.^4^ These interventions range from mobile health applications (mHealth), telemedicine, and online support networks, to digital harm reduction tools aimed at improving sexual health and reducing risky behaviors.^5^ Comparably, digital health solutions have been successful in improving health outcomes in other vulnerable populations, highlighting their potential for wide-scale adoption in marginalized groups such as sex workers.^6^ In Senegal, sex work is legal and regulated through a health policy that mandates regular STI screenings for registered sex workers.^7^ While this regulatory framework ostensibly aims to protect health, its effects are complex. Evidence shows that mandatory screening can inadvertently reinforce surveillance and exacerbate mistrust, making health promotion a potential site of control rather than empowerment.^7^ This highlights the importance of co-designing digital health solutions with sex workers to ensure that interventions are evidence-based, contextually sensitive, and do not reproduce structural harms under the guise of public health.

Moreover, DHIs have demonstrated a unique capacity to reduce public health problems among sex workers.^7^ For example, mobile health (mHealth) platforms such as the “iProtect” app have been used successfully to increase condom use and regular STI testing among sex workers in Kenya.^8^ Furthermore, online peer support groups facilitated through social media have been shown to reduce stigma and improve access to health services for male sex workers.^9^ Moreover, for instance, mobile apps can offer real-time access to information about safe sexual practices, STI prevention, and available healthcare services, which can empower sex workers to make informed decisions about their health.^8,10,11^ Telemedicine services can bridge the gap for sex workers who may face geographical or social isolation, providing virtual consultations with healthcare providers, mental health counselors, or harm reduction specialists.^12^ Additionally, digital platforms can be leveraged for tracking health outcomes and offering personalized feedback, improving adherence to treatment plans, and overall health monitoring.^13^ This review builds on these known examples by identifying the breadth of DHIs across multiple contexts and evaluating their effectiveness not only in promoting individual health outcomes but also in addressing broader social and systemic barriers. While some interventions are already established in the literature, our review expands this understanding by highlighting the need for more robust evidence and interventions specifically targeting structural issues such as criminalization, stigma, and digital exclusion.^13^ Interestingly, the literature included in this review reflects a global context, incorporating studies from diverse geographic regions to ensure a comprehensive understanding of how DHIs impact sex workers across different social, economic, and healthcare systems.

However, DHIs also have the potential to address social problems such as stigma, violence, and discrimination, which are pervasive within the sex work industry.^14,15^ Social stigma often leads to a reluctance among sex workers to seek out healthcare services for fear of being judged or criminalized.^16^ Digital tools, by offering anonymity and privacy, can help mitigate these risks, providing a safe space for sex workers to access resources without fear of exposure.^17^ These features help mitigate the fear of stigma, discrimination, and legal repercussions that often prevent sex workers from seeking in-person services. For instance, anonymous online platforms allow sex workers to ask questions about sexual health, mental health, and legal rights without disclosing their identities, thereby fostering a sense of safety and trust.^18^ Similarly, telehealth services and encrypted messaging apps provide private channels to consult healthcare providers or peer counselors, which is especially valuable in regions where sex work is criminalized or heavily stigmatized.^19^ Moreover, digital platforms can be used for peer support networks, which can foster community building and resilience among sex workers, reducing the isolation that often accompanies their work.^16,20,21^ Research has shown that online communities can provide a sense of solidarity and belonging, offering emotional support and practical advice from peers who share similar experiences.^17^ This peer support can significantly influence the well-being of sex workers by reducing feelings of isolation, enhancing self-esteem, and fostering resilience in the face of stigma and discrimination. Engaging with others who understand the unique challenges of sex work enables individuals to share coping strategies, navigate health services, and advocate for their rights, which collectively promotes mental health and a greater sense of empowerment.^22^ These platforms also serve as safe spaces where sex workers can express themselves freely without fear of judgment, which contributes to emotional healing and social connectedness.

However, despite the growing recognition of digital health's potential, there is limited research specifically focused on its application for sex workers. The limited research may be attributed to the criminalization and social stigmatization of sex work in many regions, which makes it difficult to recruit participants and fund targeted studies. Additionally, existing DHIs often adopt a one-size-fits-all approach, failing to consider the unique needs, contexts, and risks faced by diverse groups within the sex worker community, such as transgender individuals or male sex workers. Further research is needed to evaluate the effectiveness, accessibility, and cultural sensitivity of digital tools, as well as to explore sex workers’ own perspectives on technology-based health support to ensure that interventions are relevant, inclusive, and empowering.^1^ Most studies have concentrated on general public health initiatives, with only a few addressing the particular needs of sex workers. Several small-scale pilot projects have shown promising results, such as mobile apps providing real-time information on safe sex practices and local health resources, yet there remains a lack of large-scale, robust evidence to guide policy and practice.^18^ Moreover, there is a need to critically assess the potential risks associated with DHIs in this context. While digital tools can offer privacy and access to healthcare services, they may exacerbate existing inequities, particularly for those without consistent access to smartphones, reliable internet, or digital literacy skills. This digital divide can result in the exclusion of the most marginalized individuals, limiting their ability to benefit from DHIs and widening disparities in health outcomes.

This review seeks to review the roles of DHIs in enhancing the well-being of sex workers, with a particular focus on identifying the interventions available, their impact on health outcomes, and the barriers to their successful implementation. By mapping existing literature and identifying gaps, this review aimed to provide a comprehensive understanding of how digital health tools can be harnessed to reduce public health and social problems among sex workers. It also intended to highlight areas for future research and guide the development of more inclusive, effective, and ethical DHIs for sex workers.

## Methodology

Since narrative review methodology is helpful in presenting novel ideas and examining under-researched issues, it was appropriate for this article.^22^ Additionally, narrative reviews can be utilized to increase theoretical understanding by critically analyzing findings in relation to the larger body of literature.^23^ To ensure rigor, we adhered to the fundamental six phases of any review: developing the study question and objectives, searching the literature, screening articles for inclusion, evaluating the caliber of the studies, and extracting and analyzing the data.^24^ The guiding questions were:

- How do DHIs address systemic social challenges, including various forms of violence, experienced by sex workers?
- What are the limitations and possible unintended consequences of DHIs for sex workers, particularly in relation to the digital divide, surveillance, and exclusion?
- What types of DHIs are employed to support sex workers?
- How do these interventions address public health issues such as STIs, mental health, and substance abuse?
- What opportunities exist for strengthening the role of DHIs, and what barriers must be addressed to maximize their impact?

### Search strategy and study selection

**Figure 1.**
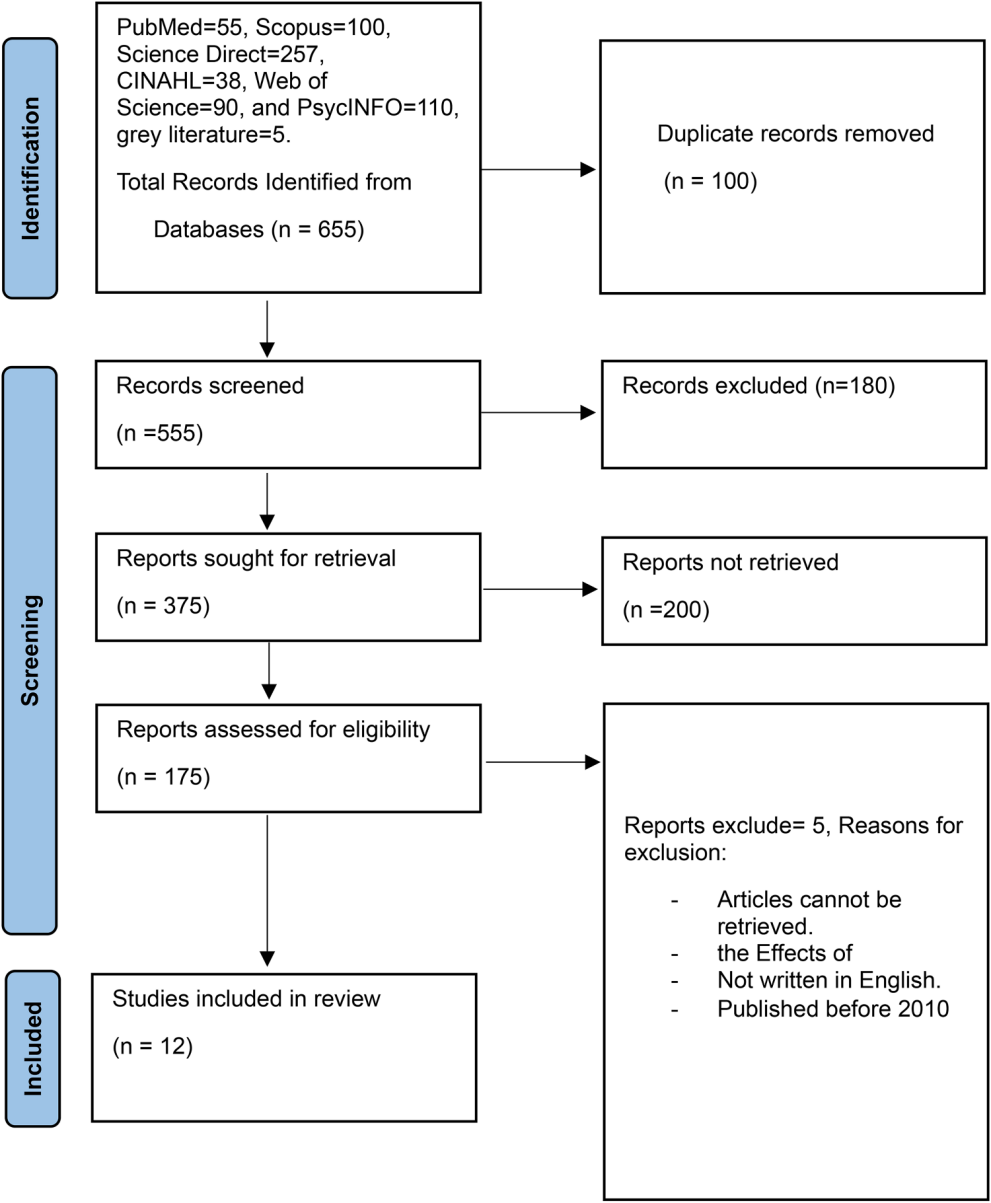
A diagram of the selection of articles.

For the main comprehensive search conducted on 10 January 2023, the databases included PubMed, CINAHL (Plus with Full Text), Scopus, Web of Science, gray literature, and PsycINFO. The search strategy, including all identified keywords and index terms, was adapted for each included database and information source. Key search terms were identified by Medical Subject Headings (MeSH), and the search strings were developed to search the databases as indicated in Table 1. These search strategies were validated by an information specialist. The reference list of all included sources of evidence was screened for additional studies. In addition, the authors have included a flowchart diagram representing the search strategy as shown in Figure 1.

**Table 1.** Search strategy.

Database	Search terms
PubMed	((“sex workers” OR “prostitutes” OR “sex work” OR “transactional sex” OR “commercial sex workers” OR “escort workers” OR “survival sex” OR “female sex workers” OR “male sex workers” OR “LGBTQ+ sex workers”) AND (“digital health” OR “eHealth” OR “mHealth” OR “telehealth” OR “health technology” OR “mobile health applications” OR “online health services” OR “virtual health platforms”) AND (“public health issues” OR “mental health” OR “substance use” OR “STIs” OR “HIV prevention” OR “healthcare access” OR “wellbeing” OR “stigma reduction” OR “psychosocial support”))
CINAHL	(“sex workers” OR “prostitutes” OR “sex work” OR “transactional sex” OR “commercial sex workers” OR “escort workers” OR “female sex workers” OR “male sex workers”) AND (“digital health” OR “mHealth” OR “eHealth” OR “telemedicine” OR “mobile apps for health” OR “health interventions”) AND (“mental health” OR “STIs” OR “HIV prevention” OR “social determinants of health”)
Scopus	TITLE-ABS-KEY ((“sex workers” OR “prostitutes” OR “commercial sex” OR “sex work” OR “transactional sex” OR “LGBTQ+ sex workers”) AND (“digital health” OR “eHealth” OR “mHealth” OR “health technology” OR “mobile applications” OR “telemedicine”) AND (“mental health” OR “STIs” OR “substance abuse” OR “psychosocial support” OR “healthcare access”))
Web of Science	TS = (“sex workers” OR “sex work” OR “commercial sex workers” OR “transactional sex”) AND TS = (“digital health” OR “telehealth” OR “eHealth” OR “mHealth” OR “mobile health interventions”) AND TS = (“HIV prevention” OR “mental health” OR “substance use” OR “stigma reduction” OR “psychosocial support”)
PsycINFO	((“sex workers” OR “sex work” OR “prostitutes” OR “commercial sex” OR “transactional sex” OR “female sex workers” OR “male sex workers”) AND (“digital health” OR “telehealth” OR “eHealth” OR “mobile health apps” OR “online health services”) AND (“mental health” OR “substance use” OR “STIs” OR “HIV prevention” OR “wellbeing” OR “stigma” OR “health access”))

### Quality assessment

To ensure methodological rigor and minimize bias, a formal quality assessment of all included studies was conducted. The Critical Appraisal Skills Programme (CASP) checklist for qualitative research was used to evaluate the credibility, relevance, and trustworthiness of each study.^25^ Each article was independently assessed by two reviewers across key domains, including clarity of research aims, appropriateness of methodology, recruitment strategy, data collection, reflexivity, ethical considerations, data analysis, and the value of the research. Studies were rated as high, moderate, or low quality based on the extent to which they met the checklist criteria. Discrepancies in ratings were resolved through discussion and, where necessary, consultation with a third reviewer. Only studies rated as moderate to high quality were included in the synthesis to ensure the robustness and reliability of findings and to reduce the risk of bias influencing the interpretation of results.

### Eligibility criteria

The inclusion criteria were determined based on the Population, Concept, and Context (PCC) framework as recommended by the Joanna Briggs Institute:

- Population: sex workers (including all genders and ages).
- Concept: DHIs are defined as the use of digital tools, technologies, or platforms to deliver health-related services or information.
- Context: public health and social issues (e.g., access to healthcare, mental health, substance use, stigma).

Study designs: all types of study designs, including qualitative, quantitative, and mixed-methods studies, as well as reviews and gray literature. Language: studies published in English. Time frame: articles published from January 2010 to December 2023 to capture recent developments in digital health technologies.

### Data extraction

One reviewer used a data extraction technique in Covidence that was created and tested by both the reviewer and another reviewer to extract data for this narrative review. Participant information, the topic under investigation, the study setting, the research techniques, and the main conclusions were all included in the retrieved data. Benefits, obstacles, facilitators, and preferences were taken into consideration when extracting the data. Table 2 offers the data extraction template.

**Table 2.** Data extraction template.

1. Study detail
2. Author
3. Country
4. Year of publication
5. Study aim
6. Study design
7. Type of data
8. Data collection
9. Data analysis
10. Study population
11. Sample size/total number of participants
12. Type of digital technology
13. Findings
14. Benefits
15. Barriers
16. Facilitators
17. Recommendations

### Data analysis

To ensure alignment with the review's aim and research question, the data were analyzed using a thematic analysis approach, presented through a narrative overview of findings.^22^ Thematic analysis allowed for the systematic identification, organization, and interpretation of patterns of meaning across the included studies. Codes were generated inductively and then grouped into broader categories, which were further refined into overarching themes through an iterative process involving all reviewers. This method enabled the interpretation of both explicit and latent content, offering a comprehensive understanding of sex workers’ experiences in relation to DHIs. In addition to the narrative, tables were used to visually illustrate the main conclusions and emerging trends across the literature. Regular team discussions were held to ensure reflexivity, consistency, and to minimize individual bias during the analysis process. This collaborative approach enhanced the credibility, transparency, and reliability of the findings.

## Results

The findings of this review are presented thematically, highlighting key areas where DHIs have influenced the well-being and public health outcomes among sex workers.

### Types of DHIs for sex workers

While the introduction provides a general overview of the potential of digital health tools for sex workers, this section expands and deepens the understanding by categorizing and analyzing the specific types of interventions employed across various global contexts. This review identified several types of DHIs that have been utilized to improve the well-being of sex workers. These include mobile health (mHealth) apps, telemedicine services, online peer support networks, harm reduction tools, SMS-based health alerts, and digital safety tools.^4,10^ mHealth apps were primarily used for sexual health education, STI prevention, and encouraging safer sex behaviors. Harm reduction tools helped manage substance use and promote safe sexual practices.^7^ SMS-based reminders reached sex workers with limited internet access, encouraging regular health checkups and STI testing.^7,8^ Digital safety tools offered real-time alerts and violence reporting systems, especially for street-based sex workers, improving their physical safety.^4,13^

### Impact on public health outcomes

DHIs positively impacted public health outcomes, particularly in areas such as STI prevention, mental health, and harm reduction.^4,18^ mHealth apps contributed to better awareness of STI prevention and safer sexual practices. The harm reduction tools helped sex workers engage in safer sexual practices and substance use management, leading to a decrease in risky behaviors and improving overall health.^4,8,9^ Moreover, SMS-based health alerts have proven effective in enhancing engagement with healthcare services, particularly for sex workers with limited or inconsistent internet access. These text-based reminders play a vital role in appointment adherence, medication follow-ups, and timely STI screening.^8,9^

Importantly, an intersectional lens reveals that the effectiveness of digital health tools is not uniform across all sex worker populations. Factors such as gender identity, race, socioeconomic status, immigration status, and level of digital literacy influence both access to and outcomes from these interventions. For example, transgender sex workers may face compounded stigma and discrimination in digital spaces, while migrant sex workers might struggle with language barriers or fear of surveillance.^19^ As such, public health interventions must be tailored to address these intersecting vulnerabilities to ensure equitable health outcomes for all individuals within the sex work community.

### Reduction of social problems (stigma, violence, and discrimination)

One of the most significant benefits of DHIs was their role in addressing social problems, particularly stigma, violence, and discrimination. Peer support networks and anonymous health platforms played a crucial role in reducing stigma by offering sex workers a space to seek healthcare and support without fear of judgment.^18^ This platform also helped foster a sense of community and empowerment, reducing isolation and social marginalization. Moreover, digital safety tools provided real-time safety monitoring and violence reporting systems, enabling sex workers to quickly access emergency services, thereby reducing their exposure to violence and harassment.^4,18^

While digital platforms have created new avenues for support and harm reduction, it is essential to acknowledge that they can also serve as sites of violence for sex workers. Online spaces are not inherently safe and may expose users to risks such as image-based sexual abuse, as highlighted by Redman and Waring.^26^ The relationship between digital technology and violence is complex; beyond privacy breaches, the misuse of digital imagery and online harassment significantly undermines the potential of these platforms to function as safe spaces. This duality must be considered when evaluating the effectiveness and safety of DHIs.

### Barriers and facilitators to implementation

While DHIs showed positive outcomes, there were several barriers to their implementation.^14^ Limited access to technology, particularly smartphones and reliable internet, hindered the effectiveness of some interventions, especially for sex workers in rural or low-income areas.^13^ Privacy and security concerns also presented significant barriers, particularly for those in regions where sex work is criminalized, as sex workers feared surveillance or data breaches.^13,14^ Despite digital exclusion being a major barrier, SMS-based interventions emerged as a promising solution for sex workers with limited internet access.^14^ These tools are cost-effective, require minimal digital literacy, and have been successfully used to deliver health reminders, promote STI testing, and provide harm reduction information, thereby increasing accessibility among marginalized groups. Furthermore, digital illiteracy in some populations limits their ability to fully engage with digital tools.

Another key barrier is searchability. Sex workers may struggle to find interventions designed for them due to limited visibility, underfunded search engine optimization, or a tradeoff between broad outreach and maintaining privacy. In addition to low digital literacy and privacy concerns, deplatforming presents a significant barrier, as sex workers are often removed from social media platforms and denied access to digital spaces essential for health information, community support, and safety as evidenced by research from the Hacking/Hustling collective.^27^

While digital tools play a critical role in enhancing safety for sex workers through features such as real-time violence alerts, location sharing, and anonymous reporting their effectiveness is increasingly threatened by legal frameworks that criminalize or restrict digital platforms. For example, legislation such as FOSTA/SESTA in the United States, which was intended to combat sex trafficking, has inadvertently led to the shutdown of platforms like Backpage that many sex workers used for screening clients and ensuring safer working conditions.^27^ As highlighted in the report Erased: The Impact of FOSTA-SESTA,^27^ such legal interventions often prioritize punitive approaches over harm reduction, undermining public health goals and stripping sex workers of vital digital safety infrastructure. This demonstrates the urgent need to align digital health strategies with legislative and policy reforms that center the rights, safety, and well-being of sex workers.

## Discussion

The narrative review aimed to explore the roles of DHIs in enhancing the well-being of sex workers and addressing public health and social issues. As demonstrated across the reviewed literature, DHIs serve as a catalyst for change by addressing both the health and systemic social challenges faced by sex workers.^28^ The findings reveal a range of digital tools, such as mHealth apps, telemedicine services, and digital safety tools, which have been utilized to enhance access to healthcare, promote safer practices, and reduce harm. These interventions not only align with the needs of sex workers but also contribute meaningfully to broader public health outcomes.^16,29^ However, digital literacy and privacy concerns, contextual barriers such as online violence, deplatforming, and shadow banning further restrict sex workers’ access to and engagement with DHIs, limiting their reach and effectiveness.

mHealth applications have been particularly effective in addressing sexual health education, STI prevention, and encouraging safer sex behaviors. For instance, their utility in promoting regular STI testing and disseminating targeted health education aligns with existing evidence that such tools enhance health literacy and health-seeking behaviors in vulnerable populations.^23^ Similarly, telemedicine platforms have provided a critical avenue for addressing mental health challenges, with studies demonstrating reductions in depression, anxiety, and substance use symptoms among sex workers who engage with remote counseling services.^24^ Conversely, the accessibility of these interventions is often hindered by technology and internet limitations, especially in rural and low-income settings.^25^ This observation is consistent with research emphasizing the persistent digital divide, which disproportionately impacts marginalized groups.^25^

DHIs have also contributed significantly to public health by reducing risky behaviors associated with substance use and unprotected sex. Tools designed for harm reduction, such as apps and SMS-based alerts, facilitated safer substance use and encouraged adherence to STI testing schedules, which ultimately improved health outcomes.^4^ Specifically, these interventions led to reductions in STI incidence, improved mental health through increased access to psychological support, and enhanced treatment adherence for individuals managing chronic conditions such as HIV. These findings resonate with literature that underscores the importance of targeted interventions for populations with limited access to traditional healthcare services.^4,18^ However, the success of these tools is contingent on their ability to address contextual barriers, such as digital literacy and privacy concerns.^14^

Beyond health outcomes, digital platforms have played a transformative role in addressing social challenges, such as stigma, violence, and discrimination. Peer support networks and anonymous health platforms offered a space for sex workers to connect, seek support, and feel empowered without fear of judgment.^4,30^ These interventions helped reduce feelings of isolation and fostered a sense of community, a finding echoed in studies that highlight the psychosocial benefits of online peer support among stigmatized groups.^16,17,24^ The introduction of digital safety tools further enhanced physical safety by providing real-time alerts and violence reporting systems, enabling sex workers to access emergency services promptly.^4^

However, the implementation of these interventions faced notable barriers. Limited access to technology and the internet, especially among sex workers in rural areas, posed a significant challenge.^14,25^ Privacy and security concerns were also prevalent, particularly in regions where sex work remains criminalized.^31^ Such concerns are well-documented in the literature, which emphasizes the need for robust data security measures to ensure user trust and engagement.^18^ Similarly, digital illiteracy among some populations limited the uptake and effectiveness of these tools, emphasizing the importance of user-centered design and training programs to enhance accessibility.^30^

The findings of this review could be further strengthened by adopting the social ecological model (SEM) as a guiding framework. The SEM emphasizes the interplay of individual, interpersonal, community, and societal factors in shaping health behaviors. Applying this model can provide a more holistic understanding of how DHIs operate within the broader context of sex workers’ lives.^32,33^ For instance, at the individual level, digital tools can enhance health literacy and self-efficacy, while at the community level, peer support networks can foster social cohesion and empowerment. At the societal level, interventions addressing stigma and discrimination can contribute to systemic change.^27^ By incorporating the SEM, future research and program development can ensure that DHIs are comprehensive, contextually relevant, and sustainable.

### Gaps in the literature and future directions

The review revealed several gaps in the current literature on DHIs for sex workers. While many studies highlight the immediate benefits of these interventions, there is a lack of longitudinal studies assessing their long-term effectiveness. Additionally, there is limited research on how intersectionality, such as race, gender identity, and socioeconomic status, impacts the effectiveness of digital health tools for different subgroups of sex workers. Ethical concerns regarding data privacy, surveillance, and the use of digital tools in environments where sex work is criminalized also need further exploration. Future research should focus on addressing these gaps, ensuring that interventions are both effective and ethically sound, and exploring the long-term impact of digital health tools on the health and well-being of sex workers.

Research underscores the importance of co-design with sex workers, ensuring that interventions are not only evidence-based but also responsive to the lived realities of the communities they intend to serve.^20^ The Hacking//Hustling collective exemplifies research led by sex workers themselves, highlighting the transformative potential of participatory and community-driven approaches in digital health innovation.

Ethical research with sex workers requires sensitivity to power dynamics, confidentiality, and respect for autonomy. In line with Brookfield's (2020) five guiding principles, which include safety, consent, anti-oppression, participation, and rights-based approaches, researchers are urged to adopt inclusive and empowering methodologies that protect participants from harm and stigma.^34^ This aligns with the growing emphasis on co-production, where sex workers are actively involved in the design and implementation of research. Such approaches not only enhance ethical integrity but also improve the relevance and impact of interventions tailored to the lived realities of sex workers.^35^

A notable gap across the reviewed literature is the lack of differentiation between subgroups of sex workers, such as street-based workers, escorts, or online-based workers. Most studies treat sex workers as a homogeneous group, without considering how the effectiveness of DHIs might vary based on the context of their work or socio-demographic characteristics.^36^ This limits our understanding of which interventions work best for specific subpopulations.

### Study limitations

This review has several limitations that should be acknowledged. First, the scope of the review was limited to studies published in English, which may have excluded valuable insights from non-English literature. The diversity of DHIs and varied contexts across regions posed challenges in synthesizing findings, as differences in technology access and legal frameworks for sex work were not uniformly addressed. Furthermore, the review did not extensively evaluate the methodological quality of included studies, which could influence the reliability of the reported outcomes. Although this review draws on global literature, it does not provide a systematic comparison of how different legal frameworks impact the implementation and effectiveness of DHIs for sex workers. However, it is important to emphasize that legal contexts, especially the criminalization of sex work, serve as structural barriers. Criminalization fosters fear of surveillance, arrest, and platform censorship, all of which can undermine trust in and access to digital health services.

## Conclusion

This narrative review found that DHIs, such as mHealth apps, telemedicine, and harm reduction tools, effectively improve healthcare access, promote safer practices, and address stigma and violence among sex workers. These tools positively impact public health outcomes by reducing STIs, managing mental health challenges, and fostering community support. However, barriers like limited digital access, privacy concerns, and digital literacy remain challenges. To address digital access limitations, partnerships with non-governmental organizations and public health agencies could facilitate the distribution of subsidized or donated smartphones and data vouchers specifically for vulnerable populations, including sex workers.

Privacy concerns can be mitigated through the development of secure, encrypted platforms that allow anonymous usage, coupled with user education on digital safety and confidentiality practices. To tackle low digital literacy, targeted digital education programs delivered in community settings or through peer-led workshops can empower sex workers to navigate digital health tools confidently and safely. By implementing these practical and inclusive strategies, DHIs can become more accessible, equitable, and effective across diverse segments of the sex worker population.
